# Testing the single-pass VOC removal efficiency of an active green wall using methyl ethyl ketone (MEK)

**DOI:** 10.1007/s11869-017-0518-4

**Published:** 2017-10-27

**Authors:** Fraser Torpy, Nicholas Clements, Max Pollinger, Andy Dengel, Isaac Mulvihill, Chuan He, Peter Irga

**Affiliations:** 1 Plants and Environmental Quality Research Group, School of Life Sciences, Faculty of Science, University of Technology Sydney, Sydney, Australia; 2 Well Living Lab, Rochester, MN USA; 3 Delos Labs, Delos, New York, NY USA; 4 General Internal Medicine, Mayo Clinic, Rochester, MN USA; 5 Delos, New York, NY USA; 6 Building Research Establishment, Watford, UK

**Keywords:** Biofiltration, Indoor air, Indoor plants, Air pollution, CADR, Phytoremediation

## Abstract

In recent years, research into the efficacy of indoor air biofiltration mechanisms, notably living green walls, has become more prevalent. Whilst green walls are often utilised within the built environment for their biophilic effects, there is little evidence demonstrating the efficacy of active green wall biofiltration for the removal of volatile organic compounds (VOCs) at concentrations found within an interior environment. The current work describes a novel approach to quantifying the VOC removal effectiveness by an active living green wall, which uses a mechanical system to force air through the substrate and plant foliage. After developing a single-pass efficiency protocol to understand the immediate effects of the system, the active green wall was installed into a 30-m^3^ chamber representative of a single room and presented with the contaminant 2-butanone (methyl ethyl ketone; MEK), a VOC commonly found in interior environments through its use in textile and plastic manufacture. Chamber inlet levels of MEK remained steady at 33.91 ± 0.541 ppbv. Utilising a forced-air system to draw the contaminated air through a green wall based on a soil-less growing medium containing activated carbon, the combined effects of substrate media and botanical component within the biofiltration system showed statistically significant VOC reduction, averaging 57% single-pass removal efficiency over multiple test procedures. These results indicate a high level of VOC removal efficiency for the active green wall biofilter tested and provide evidence that active biofiltration may aid in reducing exposure to VOCs in the indoor environment.

## Introduction

Air pollution is a major worldwide public health issue, with air pollution exposure attributed to seven million deaths globally in 2014 (World Health Organisation (W.H.O) 2014). Whilst a portion of an individual’s pollution exposure is directly linked to outdoor air (Lawin et al. 2017), a growing body of evidence identifies indoor air pollutants as having significant health impacts on humans within the built environment (Gibson et al. 2013), spaces in which contemporary urban populations spend 90% of their lives (EPA 2014). Volatile organic compounds (VOCs) are largely anthropogenic pollutants commonly associated with poor-quality indoor air (Lai et al. 2004; Lu et al. 2012; Steinemann 2015; Su et al. 2011). The health effects of VOC exposure are well studied (Rumchev et al. 2004); many VOCs are labelled as category 1 human carcinogens (Bernstein et al. 2008; Mitchell 2013). Consequently, there is a priority to reduce VOC exposure in the built environment (Ayala et al. 2012).

Whilst many filtration technologies exist for reducing indoor VOCs, including adsorption filters, photocatalytic oxidation purifiers, ozone generators, and ionisers (Ren et al. 2017; Zhang et al. 2011), such techniques are often expensive, remove a constrained range of VOCs, and can produce harmful by-products (Revah and Morgan-Sagastume 2005). The development of indoor air phytoremediation technologies may lead to an economical and sustainable departure from these conventional techniques, with potential for incorporation into the built environment for the amelioration of indoor VOC concentrations (Rodgers et al. 2012; Siswanto et al. 2016). Most research on indoor air phytoremediation has focused on potted plants (Aydogan and Montoya 2011; Irga et al. 2013; Kim et al. 2016; Orwell et al. 2006; Xu et al. 2011; Wolverton et al. 1989). This research has identified the substrate microbial community as the primary agents of VOC removal, with an ‘induction’ phase required before maximal biodegradation performance is attained (Orwell et al. 2004). However, active botanical biofiltration technology, specifically forced-air biowall systems, has received far less research. The increased plant density, vertical alignment, and high volume exposure of the plant growth media to the atmosphere are all advancements on traditional potted plant arrangements (Torpy et al. 2016). Functionally, these systems facilitate the movement of polluted air through the plant root systems and growing media and rely on a combination of plant leaves, rhizospheric microorganisms, and the chemical and physical properties of the media to adsorb or absorb pollutants from indoor air and biodegrade them in situ (Soreanu et al. 2013).

The majority of existing literature demonstrating that indoor vegetation can remove VOCs from indoor air has been conducted utilising small-scale in vitro experimental set-ups, constraining the generalisation of their findings to real-life settings (Dela Cruz et al. 2014a; Thomas et al. 2015). These experiments commonly involve injecting a single pulse of surrogate VOC (usually benzene, toluene, or formaldehyde) into a sealed, static chamber and recording the decay of VOC concentration over time and have often utilised initial VOC concentrations that are substantially higher than those found in situ (Waring 2016). It has been demonstrated that the performance of air cleaning media at high VOC concentrations cannot directly reflect their performance at the typical indoor concentration (Zhang et al. 2011), thus limiting the reliability of some previous findings.

With the development of active botanical biofiltration, there is a growing requirement to assess the functional performance of these systems and develop metrics to enable comparison to existing systems (Waring 2016). Whilst many metrics (e.g. clean air delivery rate; CADR) could be calculated from static chamber test decay curves, the chamber-to-room volume ratios utilised in many experiments are too small to be representative of in situ functional VOC removal. Additionally, it is possible that calculated VOC removal rates may be lower for experiments testing a single pulse of VOC than those using exposure to a continuous VOC levels (Dela Cruz et al. 2014b). Moreover, single-pass removal efficiency experiments may be more representative of the efficiency of a system in use, where VOCs are continuously emitted from their sources at relatively stable concentrations, thus presenting a constant pollutant input to the biofilter. Static chamber studies thus provide a test of the efficacy of a system to draw down pollutants, but do not provide information on their quantitative efficiency.

The work presented here demonstrates a methodological approach to quantifying the filtration efficiency of active botanical biofiltration systems, which could allow different systems to be objectively compared. The methodology is applied to the testing of a commercial device in a controlled, realistically sized chamber for the phytoremediation of methyl ethyl ketone (MEK) at concentrations of in situ relevance. In the current work, the combined VOC removal effects of the substrate media and botanical component were tested together, representative of the overall functional performance of the biowall in situ. Authenticated international standards were employed to enable relative methodological comparisons for future work validating the function of active green wall biofiltration.

## Methods and materials

### Biofilter

**Fig. 1:**
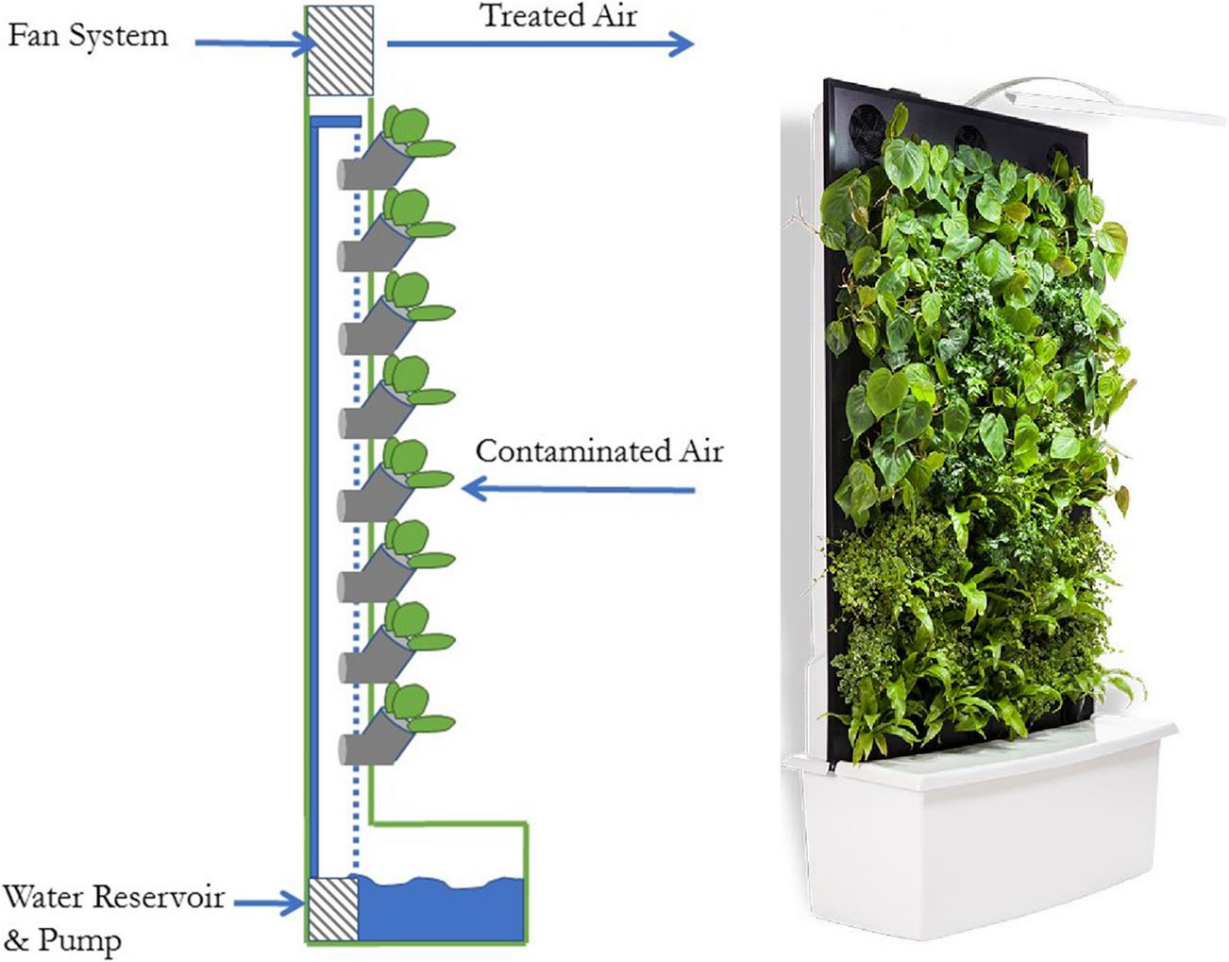
Schematic of the botanical biofiltration system tested and photograph of the fully assembled system. The plants displayed in the photograph of the botanical biofilter differ from those described in the current work

The biofiltration system used in these trials was a commercial active living wall biofilter (Naava One system, Naturvention Pty, Jyväskylä, Finland; Fig. 1), which is a free standing indoor plant wall. The system is composed of two functional components: (1) an inorganic growing media to support plant viability, along with activated carbon to assist in VOC removal, which acts as a biofilter through which ambient indoor air is drawn with an integral electric fan, and (2) the system holds 63 plants which grow horizontally from circular compartments in the front vertical face of the casing, which is 150 × 100 cm in area. The plant species used in the experimental green wall units were 18 individual *Philodendron scandens*, 13 *Philodendron scandens* ‘Brazil’, 19 *Asplenium antiquum*, and 13 *Syngonium podophyllum*. These plant species are popular in indoor plant wall arrangements and were selected on this basis alone, rather than their ability to remove VOCs. The size of the plants was such that the combined foliage completely covered the front face of the plant wall casing. The airflow rate through the plant wall was set at 50 m^3^ h^−1^, with air flowing through the foliage and substrate and into an exhaust duct at the top of the wall. In normal use, effluent air is returned to the room, whereas in the experimental system, it was ducted from the test chamber for testing. The growing media moisture content was increased to saturation twice daily, with manual watering at 05:00 and 17:00.

In order to validate the test methodology and removal performance, MEK was selected as the challenge gas in this study. MEK is a VOC that is commonly found in interior environments through its use in textile and plastic manufacture, and outgassing from printed and painted materials (Zaleski et al. 2007). MEK’s molecular weight is 72.1057, vapour pressure is 74 Torr at 20 °C, solubility in water is 24.0% at 20 °C, and Henry’s Law constant is 4.67 × 10^−5^ atm-m^3^/mol. Exposure to MEK vapour causes upper respiratory tract irritation at 100 ppmv, eye irritation at 200 ppmv, headache at 300 ppmv, and severe symptoms including paraesthesia at 300–600 ppmv (Zaleski et al. 2007). The current permissible occupational exposure limit for MEK is 200 ppmv, as an 8-h time-weighted average (Occupational Safety and Health Administration (OSHA) 2017). The odour threshold of MEK is 5 ppmv, which is well below the recommended exposure limit; thus, it is treated as a chemical with adequate warning properties (Centres of Disease Control and Prevention (CDC) 2017). It was used in this study as a VOC of concern to human health through occupational exposure and is a VOC that has received relatively little prior study. The MEK concentration used for these tests (30 ppbv) was selected to represent the relative occupational exposure concentrations likely to be found in indoor environments. Such VOC levels are nonetheless of considerable health concern, as long-term, low concentration exposure to VOCs has been linked to pulmonary problems in both mice (Wang et al. 2014) and humans (Arif and Shah 2007). MEK exposure alone shows low acute and subchronic toxicity but, when combined with other substances as is often the case in situ, it can exacerbate their toxicity (Cosnier et al. 2017). For example, it has been demonstrated that MEK facilitates the hepatotoxic effects of carbon tetrachloride (Goldstein et al. 2013).

### Chamber set-up and operation

**Fig. 2:**
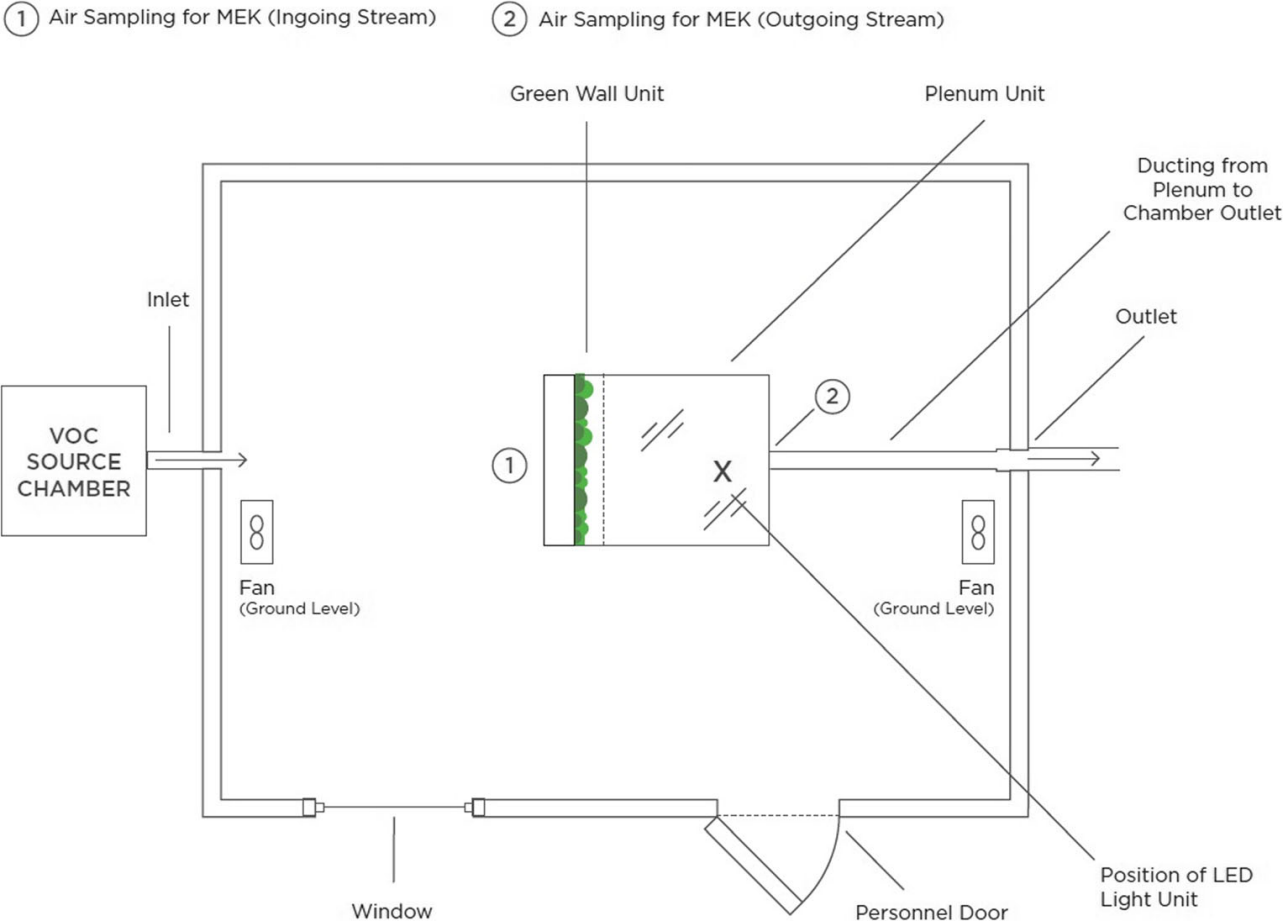
Schematic of the test chamber. VOC concentrations were monitored at both chamber inlet and outlet during the trials

The environmental chamber used for testing (Fig. 2) was built to the ‘European reference room’ specifications given in CEN/TS 16516 (CEN/TS 2013). The chamber had a volume of 30.0 m^3^ (4.0 × 3.0 × 2.5 m high) and was constructed from structural insulation panels with food grade painted steel wall and ceiling surfaces and an aluminium floor, so as to minimise VOC emissions or sorption. The chamber walls were 80 mm thick. Temperature and humidity were continuously monitored using Vaisala HMP110 Humidity and Temperature Probes (Vaisala Oyj, Helsinki, Finland) and controlled to 21.5 ± 2 °C and 37.5 ± 2.5% RH. The ventilation rate in the chamber was controlled to within 4% of the target value of 50 m^3^/s or 1.67 air changes per hour (ACH) by maintaining the airflow rate of the supply via a low-level 150-mm diameter stainless steel horizontal air discharge valve, using an orifice plate flow control system. Air was exhausted via a plenum fixed to the top of the green wall and then to a 150-mm stainless steel duct. Exhaust airflow from the green wall was monitored via TSI Venturi model No: 2017 inline venturi and a CMR Controls pressure sensor was used to set the airflow over the green wall in the chamber. These instruments were calibrated as a system using a Chell Mass Flow Meter over their full working range prior to experimentation. The control system maintained the room at + 4 Pa pressure difference relative to the outside. Chamber air mixing was accomplished using two small oscillating fans.

A LED floodlight was mounted on a stand 0.80 m from the green wall to give an average illuminance of approximately 2500 Lux (40 μmol s^−1^ m^−2^ photosynthetically active radiation) at the green wall for an 18 h day^−1^ photo-period. Lighting was controlled remotely by mobile phone signal (Naava Family Phone app, Helsinki, Finland), as were plant watering and green wall ventilation fans. An additional heat source matching the LED heat input to the room was used during the LED off periods to maintain a constant load on the ventilation system for the duration of the test.

To enable the introduction of a controlled concentration of airborne MEK, air was drawn through a separate upstream source chamber using the main chamber air handling system. Sources of MEK were trialled with the aim of providing a reasonably stable source of the compound at the required concentration in the chamber at an airflow rate of 50 m^3^ h^−1^ (i.e. 1.67 ACH in the 30 m^3^ chamber space). The MEK source used for the trials utilised an in-house wicking system, consisting of the solvent in 25 × 0.8 cm internal diameter steel tubes with 10-mm-long, 10-mm-diameter silicon sponge cord. One silicon sponge cord was used per tube. MEK was introduced to the chamber at the required level using one of two separate upstream source chambers developed and used in the course of the EC FP7 ‘ECO-SEE’ research project (Da Silva et al. 2016). One source chamber was stand-alone and used to verify the source emission rates, and the other was connected to the main chamber air handling system for doping the chamber air. The gas inlet was 0.3 m above the floor of the chamber, and the outlet was 0.3 m below the ceiling of the chamber.

### Test protocol

The experimental sequence was repeated in full three times, with new plants in the green wall unit for each sequence during step (b), followed by a 3-day acclimation period with the fresh plants before testing in step (c).

- *Chamber loss trial* (*empty green wall*). The empty green wall unit was comprised of the outer casing only, completely empty of growing medium and water. The chamber ventilation system was operated for 4 h with the MEK source on. Air sampling was performed from ingoing and outgoing gas streams (one sample from each). The chamber loss trials were the reference samples against which plant wall bioremediation activity was compared; thus, the plant wall effect includes both plant and substrate activity. We did not use a substrate only reference sample as the relative effects of substrate and botanical components of the system were not of interest in the current work.
- *Installation of fresh plants in the green wall unit.*
- *Acclimatisation period*. Green wall unit fans and watering system were operated as described previously. The system was thus operated for a period of 3 days with the MEK source on, to facilitate microbial ‘induction’ to the VOC.
- *Filtration measurement test*. Green wall unit fans and watering system were operated as described previously. The system was run for a period of 8 h with the MEK source on. Air samples were taken hourly, with sampling from ingoing and outgoing gas streams (i.e. eight samples from each).
- *Removal of plants*.
- *Chamber loss trial* (*empty green wall*). The system was run overnight with the MEK source on. Air sampling was performed from ingoing and outgoing chemical streams (one sample from each, at the end of this sequence).

### Sampling and analysis of MEK in chamber air

Active sampling of chamber air for MEK was carried out near the chamber inlet and at the outlet from the green wall test assembly using PTFE tubing, Tenax TA® sorbent tubes (length 3.5 in.; internal diameter 0.25 in.) and sampling pumps (Gilian LFS 113 DC low flow samplers) calibrated using a Mesalabs Bios Defender 520 flow meter. Typical flow rate was 200 mL/min. MEK trapped on the adsorbent tubes was quantified by automated thermal desorption and gas chromatography (Perkin Elmer Analytical TD/GC) using flame ionisation detection (FID). This analytical method is as prescribed in the International Standard BS ISO 16000-6 (BS ISO 2011). The concentration of MEK was determined using a reference calibration factor, with toluene as reference compound. Concentrations ranging from 3 to 3000 ng were spiked on to tubes and analysed for the purposes of calibration. The LOQ for MEK was 10 ng on the tube.

### Data analysis

Data are displayed as means and standard errors (SEM). Average inlet versus outlet concentrations of MEK at each hourly interval were compared using general linear model repeated-measures analysis of variance (RM ANOVA). Data were Ln transformed to improve homogeneity of variance. Control sample analyses, which compared inlet to outlet MEK concentrations without a biofilter present, were performed using a paired-samples *t* test, based on 1000 bootstrap samples. Analyses were performed using SPSS v20.0.0, IMB Corp, 2011.

To allow comparison with other biofiltration and mechanical air cleaning systems, the clean air delivery rate (CADR) of the system was calculated, which represents the clean airflow rate produced by the system, based solely on the removal of MEK. As the current work was based on pollutant reduction of a continuous VOC source rather than static chamber draw down, exponential pollutant decay rates were unavailable for the determination of CADR (see Wang et al. 2012), and these were therefore calculated as per Chen et al. (2005):

$$ \mathrm{CADR}={Q}_{\mathrm{ac}}{\eta}_{\mathrm{ac}}{E}_{\mathrm{d}} $$

where *Q*_ac_ is the air cleaner flow rate (m^3^ h^−1^), *η*_ac_ is the MEK removal efficiency (%), and *E*_d_ is the ‘short-circuiting factor’, which is equal to the inlet MEK concentration/average MEK concentration in the test chamber, which was approximated at 1 in the current experiment due to thorough mixing of the chamber atmosphere with oscillating fans.

## Results and discussion

**Fig. 3:**
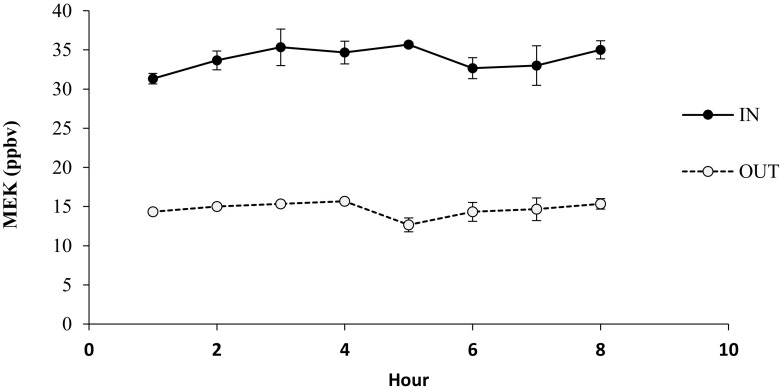
Results from three single-pass efficiency trials for biowall MEK removal. Data are means ± SE, *n =* 3

The testing conditions and results are summarised in Table 1. There was a significant difference between inlet and outlet concentrations of MEK during filtration measurement trials (Fig. 3; RM ANOVA, *F* = 220.05, *p* = 0.000), indicating that the biowall including plants had a significant capacity to reduce VOC concentrations. Inlet concentrations of 33.9 ± 0.54 ppbv of MEK were reduced to 14.7 ± 0.30 ppbv after a single pass through the biofilter, representing a single-pass removal efficiency (SPE) of 56.6 ± 0.86%. There were no significant empty chamber control MEK losses (paired-samples *t* test, bootstrap *p* = 0.634), indicating excellent chamber integrity relative to previous work, where considerable chamber losses were detected (e.g. Irga et al. 2013; Wood et al. 2002). There were no significant differences between sample time points (RM ANOVA, *F* = 2.068, *p* = 0.198), indicating a consistent VOC removal effect, with no change in efficiency over the 8-h testing period.

**Table 1:** Summary of testing conditions and results

Test ID	Target values	Trial 1	Trial 2	Trial 3
Mean chamber airflow rate, m^3^ h^−1^	50 ± 2	49.2	50.7	48.9
Mean chamber temperature, °C	21.5 ± 2	21.2	20.5	20.0
Mean chamber relative humidity, %	37.5 ± 2.5	37.0	37.3	37.0
Inlet concentration (MEK), ppb	30–40	31.63	34.25	35.88
Outlet concentration (MEK), ppb	–	13.75	15.38	14.88
Removal efficiency, %	–	56.5	55.1	58.5
CADR, m^3^ h^−1^	–	28.3	27.6	29.3

The biofiltration device produced a CADR, or airflow, from which MEK had been removed, of 28.3 or 18.9 m^3^ h^−1^ m^−2^ green wall area. A single biofilter of the type tested here would thus provide almost one air change per hour (ACH) for a room of 30 m^3^. This experiment represents the first instance that the single-pass, flow through VOC removal efficiency by an active green wall in a room-sized chamber has been performed, and is also one of few studies to document the phytoremediation of volatilised MEK.

Chen et al. (2005) compared the single-pass, large chamber pull-down removal efficiency of a prototype active botanical biofilter with several physiochemical and mechanical air cleaning devices for 17 VOCs, including MEK. Removal efficiencies for MEK ranged from 0 to 41.1%, with the tested botanical system surprisingly being found < 15% efficient for MEK removal. The system tested in the current work would appear to outperform all of those tested by Chen et al. (2005) for MEK removal efficiency, although no functionally valid comparisons can be made for CADR, as Chen et al. (2005) do not supply the filtration surface area of their test systems.

The removal of various other VOCs by active green walls has been demonstrated previously. Wang (2011) detected ~ 38% SPE for total volatile organic compounds (TVOCs) as toluene equivalents and a 90% SPE for formaldehyde, the greater magnitude of this result being attributed to dissolution of the gas in the moist filter bed of this system, the authors observing that higher moisture levels were associated with greater formaldehyde removal rates. It is apparent that VOC single-pass efficiency rates are strongly related to the solubility of the test VOC, with lower rates for nonpolar VOCs (e.g. toluene, solubility 0.052 g.100 mL^−1^), moderate rates for moderately polar VOC (e.g. MEK, 27.5 g.100 mL^−1^), and high efficiency for polar VOC removal (e.g. formaldehyde, solubility 400 g.100 mL^−1^). Lee et al. (2015) tested the SPE of an active biofiltration system for the removal of xylene, ethylbenzene, toluene, benzene (all 1 ppmv), and formaldehyde (2 ppmv), finding removal efficiencies in the range of 71.3–75% for all VOCs except benzene (39.7%) and formaldehyde (44.9%). Unlike the findings of Wang (2011), the SPEs of the various VOCs tested by Lee et al. (2015) were not correlated with their relative solubilities, which could be related to substrate differences between the systems. The relative abilities of different green wall substrates for VOC removal are still unclear and clearly require further research. It has been shown that substrates with greater organic matter content, and hence bacterial density, have a greater capacity for nonpolar VOC removal than inorganic hydroculture substrates in static potted plants (Irga et al. 2013), but this effect is unlikely to occur in active biofiltration systems unless exposed to VOCs for an extended period to allow for microbial metabolic activity to occur. Conversely, highly polar formaldehyde is removed very effectively by botanical systems based on both hydroculture (Aydogan and Montoya 2011) and soil (Kim et al. 2016). Chen et al. (2005) noted that low molecular weight, highly volatile VOCs such as formaldehyde and dichloromethane are difficult to remove using physiochemical/mechanical air cleaning systems unless specific components are added to these systems (e.g. activated alumina impregnated with potassium permanganate), and as such, it appears that biofiltration may be a practical means of mitigating these pollutants. Additionally, both the biowall system tested in the current study and the system tested by Wang (2011) contained activated charcoal within the plant growth substrate, which is known to be effective for VOC removal (Aydogan and Montoya 2011; Chen et al. 2005). Given that VOC removal by activated carbon is a physiochemical process, it is unlikely that the SPEs detected in the current work and by Wang (2011) could be replicated in systems that are solely reliant on biological processes for VOC mitigation. Activated carbon VOC saturation has been identified as a concern for long-term use in air filters (Aydogan and Montoya 2011); however, Wang’s (2011) system retained its VOC removal capacity for 300 days, and thus, this effect may not be detrimental at the low TVOC concentrations generally encountered indoors. The longevity of the substrate tested in the current work for VOC saturation was not assessed, nor was plant tolerance to VOC exposure quantified, as the botanical component of the system was replaced between test treatments. Thus, the findings presented are limited to short-term effects, and it is recommended that long-term testing be a part of future development of air phytoremediation systems.

Active biofilter air pollutant remediation trials have not been performed consistently throughout the literature, limiting the capacity to make objective comparisons between system operational components and parameters. Darlington et al. (2001) tested the inlet versus biofiltered outlet VOC concentrations for a hydroponic biofiltration system in situ in a commercial building, recording efficient VOC removal at very low inlet concentrations for toluene, o-xylene, and ethylbenzene. Variations in VOC removal efficiency were detected for both changes in temperature and ventilation rate, again indicating that a consistent and standardised approach to studying systems as used in the current work is required.

Wang et al. (2012) compared VOC removal for three airflow rates through a hydroponic botanical biofilter, detecting a correlation between VOC removal rates and increasing airflow for both formaldehyde and toluene. The magnitude of the increased rate, however, was relatively minor: the lowest airflow trials were still capable of removing significant quantities of both VOCs. Airflow rate was not varied in the current work, and the system was tested only at its operational airflow rate of 16 L s^−1^ m^−2^ green wall, which was a level determined by the green wall manufacturers to represent a practical compromise between atmosphere–substrate exposure and substrate water loss and consequent irrigation requirements. The effectiveness of the trialled airflow rate has thus not been optimised for pollutant draw down and could possibly be subject to improvement.

Plant type may also play a role in forced-air biowall VOC removal. Comparisons of the VOC removal efficiency between different plant species have been performed only for traditional potted plant systems. Liu et al. (2007) reported that the best performing plants for VOC removal were *Hemigraphis alternata*, *Tradescantia pallida*, *Hedera helix*, *Asparagus densiflorous*, *Hoya camosa*, and *Crassula portulacea*, whilst Aydogan and Montoya (2011) recorded a fourfold difference between the most (*Chrysanthemum morifolium*) and least (*H. helix*) efficient species that these authors tested for formaldehyde removal. Similarly, Wolverton and Wolverton (1993) found an almost tenfold difference in VOC removal rates between their most effective (*Nephrolepsis exaltata*, *C. morifolium*, *Phoenix roebelenii*, and *Dracaena deremensis*) and least effective (*Sansevieria trifasciata*, *Aloe barbandensis*) species amongst 33 plants tested, although these results were confounded by the use of different pot sizes amongst different plant species. Thus, whilst systems containing some species may be capable of faster VOC removal rates, all of the 120 plant species that have been tested to date (Soreanu et al. 2013) have had the capacity to remove VOCs with reasonable efficiencies (e.g. Dela Cruz et al. 2014b; Pipal et al. 2012; Wolverton et al. 1989). When considered alongside the low levels of VOCs generally found in indoor air, there appears to be no compelling evidence to suggest that VOC removal would be greatly influenced by plant choices. However, there appear to be greater inter-species differences in formaldehyde removal amongst plant species than for other VOCs, which may influence species selection if this VOC is of concern in a specific application. The mixture of plants used in the current system, which did not include any species previously observed to demonstrate very high VOC removal efficiency, was still highly effective at MEK removal.

## Conclusion and recommendation for future studies

The work presented here proposed a standardised methodological approach to test the single-pass filtration efficiency of active botanical biofiltration systems and demonstrated this approach by testing the functionality of an active green wall biofiltration system. The biofilter tested demonstrated consistent single-pass removal efficiency for the test VOC, MEK. Future research into biofiltration technology should adopt procedures that allow generalisation to real world applications, and valid comparisons to be made with other systems and operational parameters. Further, additional research is required to differentiate the removal efficiency of the substrate media and moisture content, independent from combined effects of botanical biofilters in operation.
